# Emotional Experience and Psychological Intervention of Depression Patients Based on SOM

**DOI:** 10.1155/2022/5064615

**Published:** 2022-03-24

**Authors:** Yuanyuan Zou

**Affiliations:** College of Health Management, Shangluo University, Shangluo 726000, Shaanxi, China

## Abstract

Depression is a severe mental illness with an unknown pathogenesis. Clinical diagnosis is based primarily on symptoms and does not include objective biological markers. Finding objective markers for diagnosis and treatment from imaging, on the contrary, is becoming increasingly important. The SOM (self-organizing feature mapping) model was used to identify the depression tendency of users in order to investigate the emotional experience and psychological intervention of patients with depression. On this foundation, the concept of depression index is developed further, and the relationship between depression index and the severity of depression in patients is thoroughly investigated. The system can accurately and quickly identify the depression state by applying it directly to the original EEG signals, without any preprocessing or feature extraction. When combined with traditional classifiers, the analysis and comparison results show that SOM can not only effectively select features but also improve the accuracy of depression classification. This research proposes a new research direction for deep learning in the context of large-scale big data analysis.

## 1. Introduction

Depression is a common emotional disorder with a high occurrence rate, a high recurrence rate, a high suicide rate, a high rate of disability, and a high social burden [1]. Currently, the rate of depression recognition is low, and the proportion of patients who can be identified and treated in a timely, sufficient, and adequate manner is even lower [2]. Depression is a complex negative emotional [3, 4] experience characterised by subjective distress. The most recent findings from the China Mental Health Survey are as follows. The six mental disorders (mood disorders, anxiety disorders, alcohol/drug addiction disorders, schizophrenia and related mental disorders, eating disorders, and impulse control disorders) accounted for 9.3% of the total, with mood disorders accounting for the majority of the cases. The prevalence of depression in the disorder was 3.4 percent over a lifetime and 2.1 percent over a 12-month period, respectively. Domestic and international experts and scholars have made significant progress in the study of depression in recent years [5]. These advancements include the exploration of aetiology and pathogenesis, as well as the development of new diagnostic criteria and the emergence of new and better treatments, particularly the emergence of a large number of new antidepressants, which have given patients with depression good news and enabled clinical doctors to provide more effective treatments.

SOM (self-organizing feature map) neural network [6–8] is an unsupervised learning method that imitates the characteristics of self-organizing signal processing in animal and human cerebral cortex. It can automatically extract the intrinsic important statistical features of samples according to the rules of samples. This makes it widely used in intelligent control, pattern recognition, computer vision, nonlinear optimization, signal processing, etc., and now, it has become one of the important fields of artificial intelligence research [9, 10]. According to the emotional recognition results based on EEG signals, medical staff can accurately judge the current mental state of patients and the real activity of the brain and guess the degree of their illness, so as to realize accurate nursing. Personalized nursing pays more attention to the patient's own characteristics, and a reasonable nursing plan is tailored according to the patient's condition, age, education level, etc., and comprehensive in-depth nursing is implemented to improve the recovery speed of patients. When the neural network makes a correct classification by identifying the key features that can depict brain diseases, the system is considered reasonable. On the contrary, although the final result is correctly identified, the neural network does not analyze the key features, but the peripheral factors even make decisions due to the correct identification of noise or interference. Obviously, the neural network cannot meet the medical requirements due to too high false positives.

At this time, research indicates that the mechanism of depression is primarily related to abnormal activity in emotional regulation brain regions, and a large number of studies have focused on specific brain regions and neural circuits, such as the prefrontal cortex, anterior cingulate cortex, and subcortical amygdala, hippocampus, habenular nucleus, and hypothalamus [11, 12]. Long-term use of antidepressants causes damage to the liver, kidneys, extravertebral system, and other organs, and the recurrence rate is very high after stopping antidepressants, so the psychological state of patients has not improved significantly, and patients frequently cannot receive appropriate treatment, and their prognosis is poor. The purpose of this study is to understand the emotional experience and psychological intervention of patients with depression, explore the effect of psychological intervention on their negative emotional experience, provide suggestions for clinical nursing, and provide reference for the majority of clinical medical staff to implement intervention measures for patients with depression.

## 2. Related Work

China's society is experiencing a high incidence of psychological problems, and depression has become a major social problem that plagues China. Social factors are directly related to the rising prevalence of depression. In [13], studies show that depression is a high-cost disease. Early detection and treatment, especially, early diagnosis and effective treatment by physicians in general hospitals, can reduce the course of depression and suicide and can significantly reduce functional impairment and medical resource consumption, thus reducing the total burden of depression. In [14], the study also found that the negative and rejection attitude of the public towards mental illness has changed little or even worsened over the years. Literature [15] holds that it is necessary to encourage the patient's spirit of tenacious struggle against the disease, mobilize the patient's subjective initiative to fight against the disease, and work with doctors to obtain the patient's cooperation and improve the curative effect. According to [16], identifying the harbinger of disease recurrence can encourage patients to see a doctor sooner rather than later, thereby controlling symptoms, significantly improving discharged mental patients' compliance, and effectively lowering the recurrence rate. Literature [17] examines 241 living relatives of 163 first-episode schizophrenia patients and finds that the proportion of patients with high emotional experience (50.3%) is similar to that reported by foreign countries, which shows that 54 percent of patients live in families with high emotional experience. In [18], the relatives of 56 anxiety disorder inpatients were followed for 9 months, and it was discovered that the recurrence rate of patients in the high emotional experience family group was significantly higher than that of the low emotional experience family group. In [19], the results of a study of 86 psychotic patients and their relatives revealed that patients with a lot of emotional experience had higher depression and anxiety scores, but no significant difference in psychotic symptoms. This study suggests that emotional experience may be more reliable than schizophrenia in predicting depression outcomes. According to [20], patients in the experimental group had a 9.1% one-year recurrence rate, while those in the control group had a 33.3 percent recurrence rate. The two groups differed significantly. Family intervention can help patients with schizophrenia have fewer relapses and improve their social functioning.

Early brain imaging studies based on voxel morphological measurement found that patients with unipolar depression had reduced gray matter volume, which was related to their psychopathology and cognitive dysfunction [21]. Literature [22] collected 40 subjects' emotional self-rating scale data and used Lasso regression to establish a two-category model of depression prediction. In [23], uUsing BPNN (BP neural network), the recommendation algorithm of industrial organization psychology under big data is constructed. Literature [24] has built an intermediary model, which shows that depression factors in families are influenced by family identity factors and insomnia factors. Thus, sleep quality, study, family identity, and other dimensions all have an important influence on depression, indicating that the diversity of dimensions also has a positive effect on the identification of depression. In [25], from the point of view of online healthy community users' comment analysis, this study analyzes how different factors affect users' comments in online healthy communities by building a user voting adoption model. In [26], through research, it is proved that users with mental illness in online healthy communities can get certain medical care decision information by communicating with others. Literature [27] classifies patients into different types for the purpose of using online healthy communities, analyzes the patients' network information behaviors, and finds that there are significant differences in personal health information management behaviors of patients with depression in online healthy communities. Literature [28] holds that family members with high emotional experience are more inclined to attribute the patients' symptoms to personal, internal, and controllable factors and that patients can try their best to change their problem behaviors, thus showing more negative attitudes, criticisms, and controllable behaviors.

To summarise, the emotional experience of depression patients is a multidisciplinary problem, involving medicine, sociology, and psychology. The widespread negative emotional experience of depression patients has had a significant impact on their rehabilitation and quality of life and is a roadblock in the development of health care. Psychotherapy research on emotional experience can gradually be expanded to other related fields of mental diseases, and further and deeper research can be conducted to investigate other factors that affect emotional experience and patient rehabilitation from a variety of perspectives, as well as to demonstrate the characteristics of these influencing factors in the disease progression process.

## 3. Research Method

### 3.1. Analysis of Emotional Experience of Patients with Depression

Social learning theory was put forward by American psychologist Bandura. Behavior acquisition is learned by observing and imitating others. Behavior, environment, and personal internal factors interact and influence each other, and environment plays a decisive role. And through the motivation of the group, achieve collective change and driving force. Other family members will adjust themselves to better adapt to life by observing and imitating benign behaviors in daily life.

A good family environment is conducive to the healthy development of every family member's body and mind, and it is the primary environment for one's survival. Family members have an impact on and interact with one another. The behavior, cognition, and emotional changes of one family member will influence the behavior, cognition, and emotional changes of others. The suffering that depression causes patients and their families is difficult to put into words. The emotional experiences of patients and family members have a significant impact on their rehabilitation and physical and mental health. The family emotional experience of depression can be improved, the mental health of family members can be improved, family harmony can be promoted, and depression patients' symptoms can be alleviated through targeted group counseling for family members, which can meet the actual needs of family intervention for depression patients more economically and effectively. It is a novel approach and method for depression patients' family intervention.

**Figure 1:**
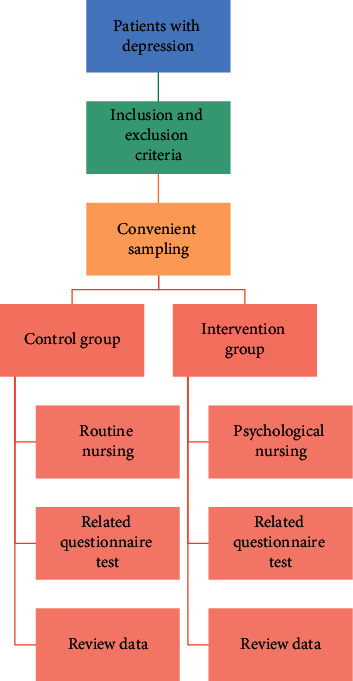
Schematic diagram of research route.

To explore the characteristics of brain activation in patients with depression under the task of positive and negative emotion induction and to test the hypothesis of emotional background insensitivity, during the experiment, each picture is presented as an experiment. The experimental process is as follows. First, the gaze point is presented for 2 seconds, reminding the subjects to pay attention to the center of the screen. Then, positive and negative emotional pictures are presented at the top of the screen, and the subjects are asked to pay attention to the emotional pictures and experience emotional arousal for 8 seconds. This study adopts the experimental design of randomized controlled study, and its specific technical route is shown in Figure 1.

After the experiment, the degree of instruction execution was checked, and all the subjects could understand the instruction and implement the instruction requirements during the experiment, including the distribution characteristics of family emotional experience of depression patients, the proportion of high emotional experience and low emotional experience, the mental health status of depression patients and their families, coping styles, and social support,. These studies are the basis of group counseling. Carry out group counseling research on family emotional experience intervention of depression patients, and design targeted group counseling implementation scheme based on the investigation of family emotional experience status of depression patients.

According to the scanning parameters and stimulus presentation mode, the statistical model is selected, the experimental variables and related time and space information are brought into the model, deconvolution is carried out according to the hemodynamic function, and the high-frequency and low-frequency images are filtered out by filtering so that the experimental design is fitted and the parameters are estimated.

Events directly affect a person's mental state, and they are also events that need certain psychological adaptation in individual life, including positive events and negative events. The former refers to events that trigger positive emotions of individuals, while the latter refers to events that trigger negative emotions of individuals. It is found that the frequency and severity of life events within two years are related to the onset of mental disorders. Negative life events and independent events may be important factors for the occurrence or recurrence of depression, while positive life events are obviously related to the occurrence or recurrence of mania. On the one hand, it shows that life events may be one of the causes of depression. On the other hand, it may be that patients' habitual defense style and its characteristics make them experience some negative life events more easily and actively than ordinary people, such as lovelorn or friends turning against each other.

### 3.2. Psychological Intervention of Depression Patients Based on SOM

As a subconscious response to conflict, a defense mechanism is a set of habitual adaptive behaviors that people use to avoid and relieve mental stress such as anxiety when confronted with a variety of frustrating situations. Patients with depression have different psychological defense styles, according to studies. Neurosis patients, including neurotic depression patients, use immature defense mechanisms more than mature defense mechanisms, according to a comprehensive study [21]. Qualitative research is sensitive to patients' subjective feelings and worldviews, which can elaborate their real experiences of diseases and health. Qualitative research encapsulates the overall concept of nursing and allows nurses to understand and feel the inner world of patients on a deeper level.

SOFM has two characteristics similar to human brain information mapping.

**Figure 2:**
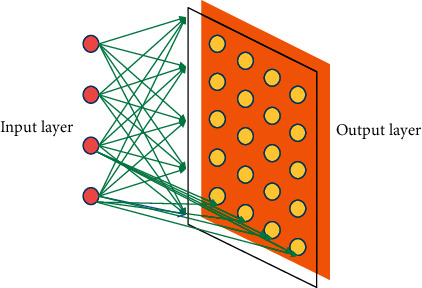
SOFM mapping model.

First, the topological mapping structure is not realized by the movement and reorganization of neurons, but a topological structure formed by each neuron in different excited states. Secondly, the formation of this topological mapping structure has the characteristics of self-organization [10]. The topological structure of the network is introduced into SOFM, and the concept of changing neighborhood is further introduced into the structure to simulate the side inhibition phenomenon in the biological neural network, so as to realize the self-organizing characteristics of the network. The SOFM mapping model is shown in Figure 2.

The network consists of an input layer and an output layer. The input layer receives signal input, and the output layer is a two-dimensional grid, which maps the output of network signals by self-organization. Each node in the two-dimensional plane grid is connected with the source node of the input layer. Each neuron in the output layer makes competitive selection in learning. The winning neuron not only strengthens itself but also drives the neighboring neurons around it to strengthen accordingly. Functionally, it can connect the change rules of individual neurons with the group change rules of one layer of neurons. After learning the network, the spatial distribution of the connection weight vectors among neurons in the output layer can correctly reflect the approximate distribution of the input pattern space.

PC (principal construct) projection is the intermediate variable of PC analysis algorithm. It is the projection matrix from the original large sample data to small samples. This method not only reduces the computation of the later algorithm but also retains the original features. Consider the projection of the sample on the PC:

(1) $$\begin{array}{c} \eta_{d}=\frac{\lambda_{1}+\lambda_{2}+\cdots +\lambda_{d}}{\sum_{i=1}^{n}\lambda_{i}},\\ y_{mi}=x_{m}p_{i}=\sum_{k=1}^{n}x_{mk}p_{ki}. \end{array}$$

It can be seen from the above formula that the projection of each data point in the original dataset on the PC is a linear combination of all the original features, and the effect of each feature on the new features can be expressed by the elements multiplied by it in the projection matrix. Therefore, the projection matrix can also be expressed as

(2) $$\begin{array}{c} C={\left[v_{1},v_{2},\cdots ,v_{n}\right]}^{T},v_{i}=\left\{p_{i1},p_{i2},\cdots ,p_{i\;d}\right\}. \end{array}$$

For each original feature, there is a line component corresponding to it, and the correlation between original features is closely related to the similarity of line components. Therefore, the original data *X*_*m*×*n*_ are converted into a projection matrix *C*_*n*×*d*_ by PC projection, the sample size of the original data is reduced from *m* to *d*, and the original feature number is maintained.

The learned “distributed feature representation” vector is mapped to the sample tag space by the full connection layer, and the tag $\bar{y}$ of Weibo content text, that is, positive, neutral, and negative probability distribution, is discriminated. The calculation formula is as follows:

(3) $$\begin{array}{c} p\left(y|s\right)=soft\max\left(w\cdot v^{*}+b^{*}\right),\\ \bar{y}=\arg\max p\left(y|s\right). \end{array}$$

Among them, *y* ∈ *R* is the true emotional tag of Weibo content, which is expressed by unique heat coding. $\bar{y}\in R$ is the emotional tag vector of Weibo content obtained through training. For each predicted emotional category probability, the tag with the highest probability is selected as the output.

When judging the depression tendency of online users from the perspective of text analysis, the depression index of users is calculated by the proportion of depression Weibo to the total Weibo number of users, so as to measure the depression tendency of users in a certain period of time. A depression index based on online healthy community is proposed, and the calculation formula is as follows:

(4) $$\begin{array}{c} DI=\frac{N_{cd}}{2N_{ct}}+\frac{N_{md}}{2N_{mt}}, \end{array}$$

where *N*_*cd*_ refers to the number of Weibo posts with depression tendency published by users in other accounts within a certain period of time, *N*_*ct*_ refers to the number of Weibo posts published by users in other accounts within a certain period of time, *N*_*md*_ refers to the number of Weibo posts with depression tendency distributed and forwarded by users in personal accounts within a certain period of time, *N*_*mt*_ refers to the number of Weibo posts distributed and forwarded by users in personal accounts within a certain period of time, and *DI* is the depression index.

The time complexity of the whole sub-SOFM is calculated as follows:

(5) $$\begin{array}{c} O\left(S\left(N,L\right)\right)=O\left(\sum_{l=1}^{d}n_{l-1}\cdot s_{l}^{2}\cdot n_{l}\cdot m_{l}^{2}\right), \end{array}$$

where *l* is the index of convolution layer and *d* is the depth, *n*_*l*_ is the number (also called width) of filters in the *l*th layer, *n*_*l*−1_ represents the number of input channels in the *l*th layer, and *s*_*l*_^2^  and  *m*_*l*_^2^ represent the spatial size of the filter and the size of the output feature map, respectively.

LSTM (long short-term memory) network is a special RNN (recurrent neural network), which can learn long-term dependent information. Through a special design, long-term information can be remembered. The core idea of LSTM is similar to a conveyor belt. The characteristic information is placed on the conveyor belt, and there is only a small amount of linear interaction so that the information can circulate on it and remain unchanged. LSTM has a well-designed structure called “gate” to remove or add information.

**Figure 3:**
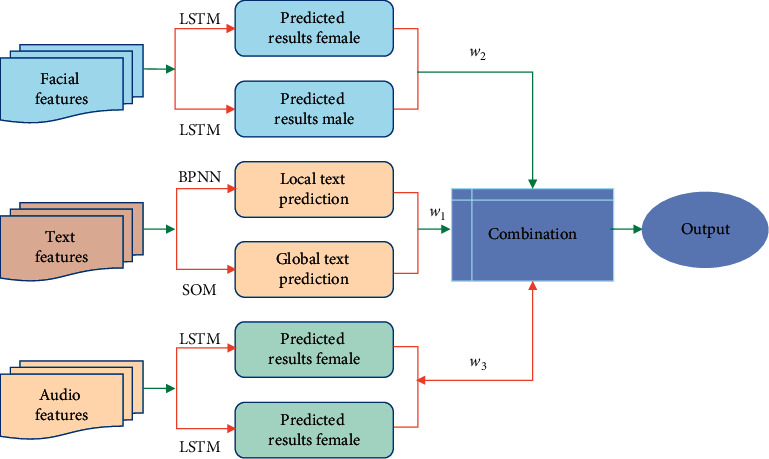
Comprehensive model.

Besides text information, there are other types of feature information that can be used in the analysis of depression tendency prediction to help improve the recognition rate. Because these features are time-sequential and change with the interview, this section uses LSTM to study the audio features of each time stamp. Finally, combining the prediction results of the three features, the following prediction collaborative classification prediction models are given, as shown in Figure 3.

The predicted results of the test set based on these three characteristics are brought into the function expression, and the results between 0 and 1 are obtained. If the calculated result is greater than or equal to 0.5, it will be counted as 1, and if it is less than 0.5, it will be counted as 0.

## 4. Results' Analysis and Discussion

**Figure 4:**
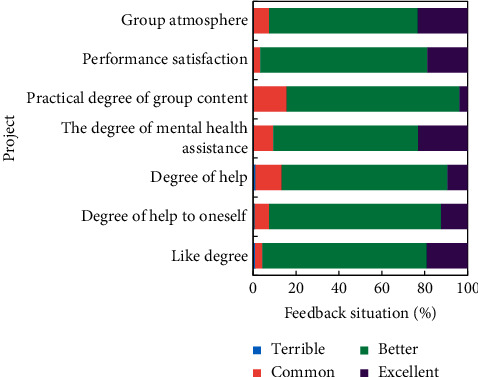
Psychological intervention effect feedback.

After experiencing the emotional group counseling, members fill in the feedback form of group counseling activities and evaluate the whole group counseling. All members feel that group counseling has a lot of gains, as shown in Figure 4.

**Figure 5:**
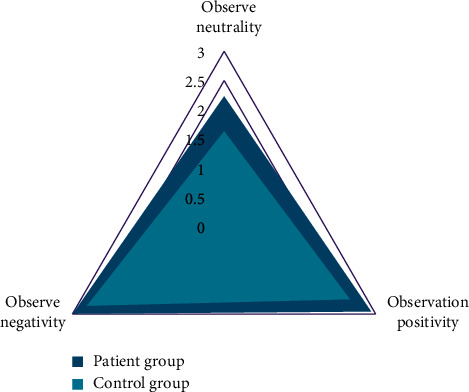
Awakening degree evaluation.

It can be seen that the psychological intervention activities of the group members (including the arrangement of psychological intervention game activities and group atmosphere) as well as the group members themselves (including self-expression, satisfaction, and harvest, among other things) have been greatly affirmed. Excel calculates the arousal rating of depression patients and normal CG emotions (1–4 grades, representing “almost no feeling,” “weak,” “strong,” and “very strong”), as well as the average value for each group. There was no significant difference in arousal degree evaluation of neutral, positive, and negative emotional materials and CG in patients with depression, according to the findings (Figure 5). The arousal degree evaluation of positive and negative emotional materials is consistent, and it is higher than the arousal degree evaluation of neutral emotional materials.

**Figure 6:**
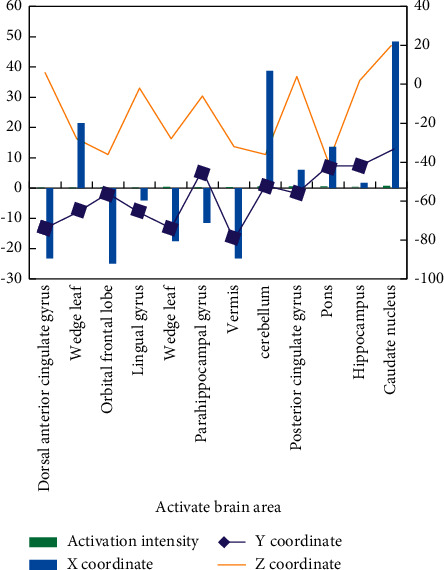
Localization and intensity of brain activation difference in positive emotion evaluation task.

The images collected at different time points are corrected at the same time point. Due to the differences of anatomical structures of different individuals, the functional images of individuals are registered with templates, and the brains of individuals are registered into the standard MNI space for functional localization of activation areas. Gaussian smoothing adopts the full width at half maximum of 8 mm to improve the signal-to-noise ratio and make the smoothed image obey the nature of random Gaussian field. For positive emotional stimulation, the differences of brain activation areas induced by two groups of subjects are shown in Figure 6.

**Figure 7:**
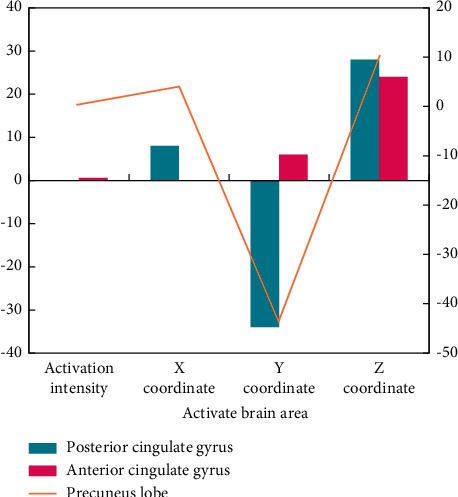
Differences of brain activation differences in negative emotion evaluation tasks.

Independent double sample *T* test was used to compare the brain activation areas and their intensity of emotional stimulation materials with a different potency in depression group and normal CG emotional evaluation task. For negative emotional stimulation, the differences of brain activation areas induced by two groups of subjects are shown in Figure 7.

The differences in brain activation of negative emotional stimulus materials between the two groups are as follows: the activation of right posterior cingulate gyrus, anterior cingulate gyrus, and right anterior wedge lobe in depression group are less than that in normal CG. The descriptive statistics of behavioral results show that the arousal degree of positive, medium, and negative emotional materials in the patient group is lower than CG, in which the arousal degree of positive and negative emotions is consistent and higher than that of neutral emotions, and the arousal degree of positive emotions is slightly lower than that of negative emotions.

Only the activated and weakened brain regions were discovered in the patient group when positive emotion induction was used, and only the activated and enhanced brain regions were discovered when negative emotion induction was used. Negative feelings activate the brain more than neutral or positive feelings. Patients with depression may have a negative bias in their emotional responses, but this hypothesis must be tested against normal CG. The anterior cingulate gyrus was activated in normal people under both positive and negative emotional conditions, which is consistent with the previously reported nonselective activation of anterior cingulate gyrus potency [18]. Dorsal cognitive control system includes the anterior cingulate gyrus. The anterior cingulate gyrus and the prefrontal cortex are jointly responsible for cognitive control, with the prefrontal cortex performing the majority of the control process and the anterior cingulate gyrus determining whether the control process needs to be changed based on the degree of reaction conflicts and errors.

It was found that the activation of anterior and posterior cingulate gyrus and right anterior cuneiform lobe was weakened under the condition of negative emotion induction in patients with depression. When comparing the positive and negative emotional evoked conditions of depression patients horizontally, it is found that these areas are not activated compared with neutral conditions. Therefore, it is speculated that the activation and weakening of these areas is not caused by emotional valence, but there is abnormality in the level of nerve spontaneous activity or basic activity. This part of the study did not make a deeper discussion about it.

**Figure 8:**
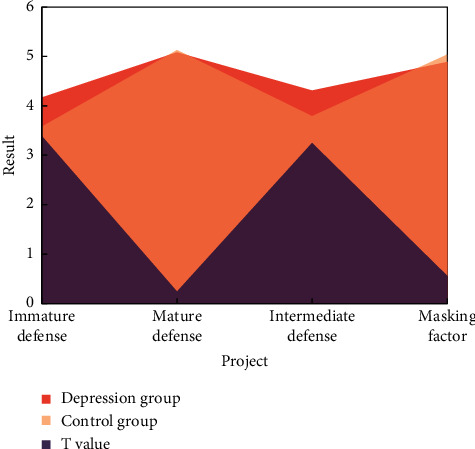
Comparison of the results of two groups of defense style questionnaires.

As can be seen from Figure 8, the immature defense style and intermediate defense style of depression group are significantly higher than those of CG, but there is no significant difference between mature defense style and masking factor.

Great changes in the environment are the source of stress for many people. When the stimulus event breaks the balance and load capacity of the organism or exceeds the individual's ability, it will be reflected in stress. These stimulus events include all kinds of situations from outside or inside, collectively referred to as stressors. Researchers have found many ways to study the relationship between life events and health. It is found that the frequency and severity of life events within two years are related to the onset of mental disorders. Negative life events and independent events may be important factors for the occurrence or recurrence of depression, while positive life events are obviously related to the occurrence or recurrence of mania. The results show that the frequency of negative life events and the total intensity of life events in depression group are higher than those in CG, and there are significant differences. This shows that, with the increase of the frequency of negative life events and the total intensity of life events, the possibility of individuals suffering from depression will increase.

Some people in the CG have a higher frequency of negative life events and a higher total intensity of life events, whereas some people in the depression group have a lower frequency of negative life events and a lower total intensity of life events, suggesting that the occurrence of negative life events can help us predict or explain the occurrence of depression in general. Any event may have different meanings for different people. The same life events and different cognitive styles will have different effects on individuals. As some scholars have pointed out, life events can affect mental health in two ways: one is direct influence and the other is indirect influence through intermediary variables.

In this study, there is no significant difference between the mature defense mechanisms of depression group and CG, which also show that the defense mechanisms are mixed and not used independently. Even in depression patients, mature defense mechanisms will be used, but the frequency that depression patients may use immature and intermediate defense mechanisms is much higher than that of them. Instead, take these three defense mechanisms as a whole. Psychological problems such as depression are not caused by the obstacles in the use of certain defense mechanisms, but related to the imbalance in the use of immature defense mechanisms, intermediate defense mechanisms, and mature defense mechanisms.

**Figure 9:**
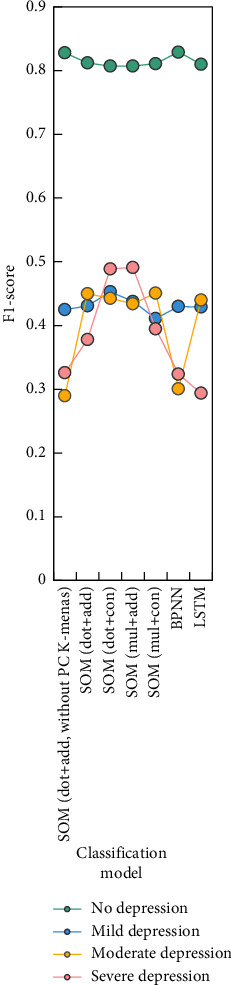
Classification of each depression level F1-score.

The F1-score of four variants of SOM and the control model in different depression grades are shown in Figure 9.

The classification performance of SOM in this study has obvious advantages over CG. Compared with other models, SOM (especially mul + add) can better and more accurately identify people with severe depression, which is very valuable in the screening of depression. Therefore, by comparing with the control model, it can be seen that the deep neural network model designed in this study shows a good classification ability, and the introduction of factor decomposition machine also plays a significant role in optimizing the network structure.

**Figure 10:**
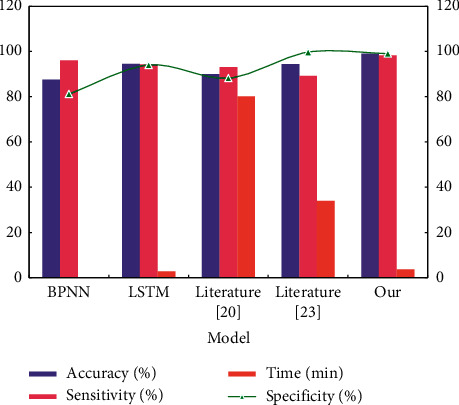
Comparison of classification performance of correlation methods.

On the same dataset (MPHCs emotional experience G data), different classifiers are used to classify depression, and the classification performance indicators are shown in Figure 10.

Figure 10 shows that the classifier proposed in this study is the best in all classification indexes, and the higher sensitivity and specificity also indicate that the classifier can effectively screen out not only depressed patients but also normal people.

According to psychologists, the narrator's process of putting past fragments and scattered experiences into complete stories is a way for the narrator to actively give meaning to changes in his or her life and bring order to disorder, which is therapeutic for the narrator. Hospital administrators should allocate medical human resources appropriately so that medical staff have enough time to sit down and communicate with patients, provide appropriate opportunities for patients to talk about their inner pain, patiently listen to their inner voice when they need it, provide emotional comfort when they are in pain, and truly enter their hearts and earn trust. They walk alongside them to help them overcome depression and pain, stay away from death's edge, and rediscover the meaning and value of life.

## 5. Conclusion

This study proposes a research method of emotional experience and psychological intervention in patients with depression based on SOM. In the face of depression-scale big data, the SOM algorithm of PC projection automatically selects the optimal feature subset on the basis of retaining the original feature space, thereby eliminating redundant information in depression-scale big data. The identification of the user's depression emotional state is divided into the judgment of the depression emotional tendency of a single text and the judgment of the user's depression state to measure the user's depression and depression. Interpretation results of the complexity of the SOM show that the classifier effectively captures the intrinsic characteristics of depression. The abnormal emotion regulation in patients with depression is mainly manifested in two aspects: one is that it is difficult to use cognitive resources needed to enhance positive emotions through cognitive reappraisal, and the cognitive control ability declines; secondly, cognitive reappraisal weakens negative emotions. It is difficult to call the required cognitive resources, and cognitive control ability declines, but it also fails to effectively reduce the activation of brain regions related to emotional evaluation.

## Data Availability

The data used to support the findings of this study are included within the article.
